# Distinct spatial patterns of perivascular spaces enlargement for multiple and Co-existing pathologies of cognitive impairment

**DOI:** 10.1016/j.tjpad.2026.100631

**Published:** 2026-07-01

**Authors:** Woosik Kim, Yejin Hwang, Yelim Yang, Min Gyeong Kim, Hyemin Jang, Seung Hong Choi, Joon-Kyung Seong, Roh-Eul Yoo, Wha Jin Lee

**Affiliations:** a School of Biomedical Engineering, Korea University, 145, Anam-ro, Seongbuk-gu, Seoul, South Korea; b AI Imaging Research Center, NeuroXT, Inc., 48, Achasan-ro 17-gil, Seongdong-gu, Seoul, South Korea; c Department of Radiology, Seoul National University College of Medicine, 101, Daehangno, Jongno-gu, Seoul, South Korea; d Department of Neurology, Asan Medical Center, University of Ulsan College of Medicine, 88, Olympic-ro 43-gil, Songpa-gu, Seoul, South Korea; e Department of Radiology, Seoul National University Hospital, 101, Daehangno, Jongno-gu, Seoul, South Korea; f Department of Artificial Intelligence, Korea University, 145, Anam-ro, Seongbuk-gu, Seoul, South Korea

**Keywords:** Alzheimer’s disease, Cerebral small vessel disease, Glymphatic dysfunction, MRI segmentation, Perivascular spaces

## Abstract

**Background:**

This study examines how amyloid-β and vascular pathology independently and jointly relate to regional perivascular space (PVS) burden on T2-weighted MRI.

**Methods:**

In 307 cognitively impaired participants retrospectively identified from the Seoul National University dementia cohort, PVS were automatically quantified in the basal ganglia (BG) and lobar white matter regions from 2D T2-weighted MRI. Amyloid-β and vascular pathology positivity were defined by [18F]Florbetaben PET and small vessel disease markers. Group differences among pathology-negative (*n*=36), vascular-only (*n*=106), amyloid-only (*n*=48), and mixed (*n*=117) subgroups, as well as amyloid–vascular interactions, were assessed using analysis of covariance and multivariable linear regression.

**Results:**

BG PVS were greater in participants with vascular burden than in pathology-negative participants (*F*=26.97, *p*<0.001; Cohen's *d*=1.28), independent of amyloid-β. Lobar PVS were higher in single-pathology than pathology-negative participants across the parietal, temporal, and occipital regions (*F*=8.25–18.04, Cohen's *d*=0.66–0.98, all *p*≤0.014), with no additional increase in the mixed group. Greater amyloid-β retention was associated with parietal, temporal, and occipital PVS in VB− participants (*β* [95% CI]=0.55 [0.19, 0.92], 0.69 [0.35, 1.02], 0.40 [0.16, 0.64], respectively). Among AB− participants, vascular burden was associated with BG PVS (*β* [95% CI]=0.65 [0.40, 0.89]). Multivariable regression demonstrated less-than-additive AB×VB interactions in parietal, temporal, and occipital PVS (*β* [95% CI]=−0.65 [−1.08, −0.23], −0.74 [−1.13, −0.35], −0.42 [−0.70, −0.14], respectively).

**Conclusions:**

Distinct regional PVS patterns reflect spatially selective and severity-dependent glymphatic-related structural alterations associated with amyloid-β and vascular pathologies, supporting PVS as a quantitative imaging biomarker for disentangling mixed pathways of cognitive impairment.

## Highlights

• Quantified regional PVS from thick-slice 2D T2 MRI using automated segmentation.
• Basal Ganglia PVS linked predominantly to vascular pathology.
• Lobar WM PVS associated with amyloid-β and vascular pathology.
• Amyloid-vascular effects on PVS burden were less-than-additive.
• Spatial PVS patterns disentangle mixed pathways of cognitive impairment.

## 1 Introduction

Perivascular spaces (PVS) are fluid-filled compartments that surround penetrating cerebral vessels and serve as essential conduits for facilitating interstitial fluid drainage and waste clearance in the brain’s glymphatic system [[1], [2], [3]]. Although their underlying pathophysiology remains elusive, PVS are known to dilate when glymphatic clearance pathways are impaired, and appear as small, tubular, cerebrospinal fluid (CSF) isointense signals in structural magnetic resonance imaging (MRI) [1,4]. Hence, PVS have emerged as a powerful neuroimaging biomarker strongly associated with glymphatic dysfunctions, which have been implicated in the etiology of a range of neurodegenerative and cerebrovascular diseases, including but not limited to cerebral small vessel disease (SVD), Alzheimer’s disease (AD), and cerebral amyloid angiopathy (CAA) [1,[5], [6], [7], [8]]. More importantly, PVS enlargement and morphology have often varied by anatomical location and disease contexts, raising the possibility that spatial PVS patterns may be specific to varying pathologies. For instance, increased PVS enlargement in the basal ganglia (BG) has shown consistent associations to hypertensive arteriopathy and other SVD markers such as white matter hyperintensities (WMH) or cerebral microbleeds (CMB) [[8], [9], [10]]. On the other hand, PVS enlargement in the centrum semiovale (CSO) is more frequently linked to impairment in amyloid-β (Aβ) clearance in AD and CAA [[11], [12], [13], [14]]. Furthermore, while the temporal and occipital regions have been underrepresented in most PVS studies, they are increasingly recognized as key sites for AD-related alterations in the glymphatic system [6].

Despite growing interests in the clinical significance of PVS enlargement, most existing studies utilize manual visual rating schemes, which involve quantifying PVS severity based on counts on predefined MRI slices [15]. While these rating scales provide conveniently robust means of assessing overall PVS severity in large regions, they lack the spatial specificity and thus may not be suitable for more detailed region-specific analyses. Moreover, recent studies have shed light on the fact that quantitative measures of PVS enlargement are more sensitive to subtle regional glymphatic alterations associated with various pathologies [[16], [17], [18]]. However, leveraging quantitative measures of PVS necessitates accurate, reliable and reproducible segmentations of PVS from structural MRIs. Manual segmentations, while theoretically precise, are prohibitively time-consuming and inherently vulnerable to inter- and intra-rater variability, underscoring the demand for studies utilizing automated segmentation pipelines [19,20]. Several automated approaches for PVS segmentation have been proposed; however, applying these methods to T2-weighted images – particularly those acquired with thick slices that are common in clinical settings – remains challenging for accurate PVS quantifications [[19], [20], [21], [22]].

While prior studies have demonstrated alterations in PVS dilation in a range of neurological conditions – using both visual rating scales and automated quantifications – most have remained restricted to broad anatomical regions, typically limited in comparisons of BG and CSO WM [[23], [24], [25], [26]]. Yet, given the selective vulnerability of specific lobar regions to different pathological processes, such as temporal lobe involvement in AD and CAA, a more comprehensive characterization of spatial dynamics of PVS may be crucial for uncovering pathology-specific regional glymphatic disruptions [6,27,28]. In addition, far fewer studies have systematically explored how these regional PVS patterns differ across multiple pathologies within the same analytical framework, making it difficult to discern shared or isolated spatial effects of PVS enlargement in mixed pathologies [10,14]. Moreover, PVS enlargement is not only a reflection of pathological presence, but also closely related to other biomarkers of pathological severity, such as WMH Fazekas scales or Aβ burden, yet few studies have examined how the spatial patterns of PVS differ across severity stages in coexisting pathologies and are also limited by nominal rating schemes [1,9,10]. In this context, continuous and regionally resolved quantification of PVS may provide greater sensitivity for capturing both the spatial topography and severity progression of PVS across mixed pathologies. Additionally, such quantitative approach could further solidify the potential of PVS for use as an imaging biomarker across varying neurological conditions.

Hence, we aim to quantitatively assess regional and severity-based alterations in enlarged PVS burden across both AD and vascular dementia (VD) cohorts — two conditions in which impaired glymphatic function and small vessel pathology are implicated through overlapping yet distinct mechanisms [[29], [30], [31]]. To enable this in clinical settings, we introduce a fully automated pipeline tailored specifically to thick-slice 2D T2-weighted MRI. We then examined region-specific PVS burden for each pathology and its associations with the severity of other pathological markers. By combining lobar anatomical parcellations with stratified pathological imaging markers, our approach enables a more detailed exploration of the spatial and severity-based patterns of enlarged PVS across various underlying disease processes.

## 2 Methods

### 2.1 Study participants

378 consecutive patients with suspected Alzheimer’s clinical syndrome or vascular cognitive impairment, who underwent a dedicated non-contrast MR and [^18^F]Florbetaben (FBB) positron emission tomography (PET) imaging for the evaluation of cognitive impairment between January 2021 and January 2023, were selected from our radiology report database. This retrospective cohort study was approved by the institutional review board of the Seoul National University Hospital, with the requirement for informed consent waived (IRB No. 2311-048-1482). The study protocol was performed in accordance with the Declaration of Helsinki. A total of 71 patients were excluded based on the following criteria: 1) MRI scans with insufficient quality due to low resolution, excessive motion artifacts, or image noise (*n*=12); 2) failure in axial-plane image reconstruction during preprocessing (*n*=54); and 3) absence of standardized cognitive function test results (*n*=5). (Supplementary Fig. 1) Cognitive function was assessed using the Korean version of the Mini-Mental State Examination (MMSE-KC) [32,33] and Clinical Dementia Rating (CDR) [34].

**Fig. 1:**
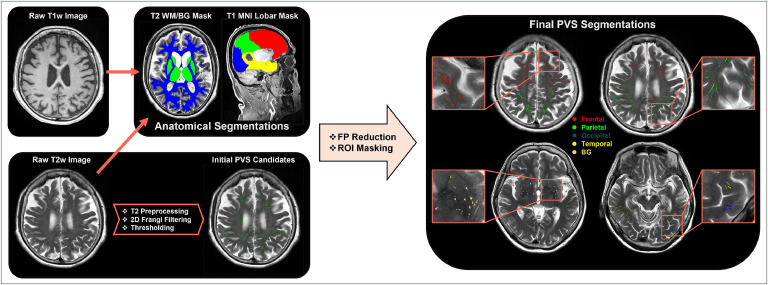
PVS Segmentation Pipeline Overview. Raw T2-weighted and T1-weighted images are used to create anatomical segmentations for ROI masks using SynthSeg 2.0 and Hammersmith n30r95 atlases respectively. Initial PVS candidates are defined by thresholding vessel probability maps obtained via 2D Frangi filtering of preprocessed T2-weighted images. FPs in initial candidates are reduced by applying size and linearity thresholds and the remaining segmentations are masked to classify into BG, Frontal, Parietal, Temporal, and Occipital WM ROIs.

### 2.2 MRI and PET imaging protocols

All MRI scans were performed on a 3.0T Siemens Skyra scanner using a dedicated dementia MRI protocol. The protocol included 3D T1W magnetization-prepared rapid acquisition gradient echo (MPRAGE) imaging, axial T2-weighted imaging, 3D T2 Fluid-Attenuated Inversion Recovery (FLAIR) imaging, susceptibility-weighted imaging (SWI), and diffusion tensor imaging (DTI). Specific imaging parameters for all sequences are provided in Supplementary Table 1.

All patients underwent a 20 min positron emission scan at 90 min after the bolus intravenous injection of FBB 296 MBq (8 mCi) using dedicated PET/CT scanners (Biograph mCT40 or mCT64, Siemens Healthcare, Germany). CT scan was used for attenuation correction, followed by an emission scan of the brain. PET images were reconstructed on 400×400 image sizes with a 1×1×1.5 mm voxel size. Images were reconstructed with ordered subset expectation maximization with 24 subsets and 6 iteration numbers. Post-reconstruction Gaussian filter (full width at half maximum 2 mm) was applied.

### 2.3 MRI image processing

Acquired T2-weighted TSE MRIs underwent N4-bias field correction, median filter noise reduction and min-max intensity normalization to reduce variability amongst images and enhance tissue contrast for consistent segmentations. To perform native anatomical segmentations on T2-weighted images, SynthSeg v2.0 was applied to normalized images as it provided robust BG and WM segmentations regardless of varying slice thickness or voxel size [35]. The Hammersmith n30r95 in MNI standard space was transferred to native T2-weighted space by using patient’s corresponding T1-weighted images as transformation mediators [[36], [37], [38], [39]]. The transformed Hammersmith atlas was used to further parcellate the WM mask into frontal, parietal, temporal, and occipital lobar regions-of-interest (ROI). All image registration and transformation processes were achieved using the Advanced Normalization Tools (ANTs) python library [40].

### 2.4 PET image processing

All FBB PET images were co-registered with their respective T1-weighted images using the FreeSurfer (FS, www.surfer.nmr.mgh.harvard.edu, version 7.2) toolbox [[41], [42], [43], [44]]. Voxel-based partial volume correction (PVC) was applied to the co-registered PET images. Standardized uptake value ratios (SUVR) were computed from resulting preprocessed PET images using the cerebellar cortex as the reference region to account for inter-subject variability. Global SUVR was calculated as arithmetic means of voxel values assigned to frontal, lateral parietal, lateral temporal, and anterior and posterior cingulate cortices [45,46].

### 2.5 PVS candidates segmentation

PVS appear as elongated CSF-isointense structures within the cerebral parenchyma of T2-weighted images [47]. Hence, potential PVS candidates were segmented by applying a 2D Frangi filter on each axial slice of the preprocessed T2-weighted images [[48], [49], [50]]. A slice-wise 2D filtering approach was used to ensure consistent extraction from large axial thicknesses of our dataset. Resulting vessel probability maps of the filter were then applied with a threshold to create a binary segmentation mask of potential PVS candidates. Filtering and threshold parameters were optimized through visual inspection of two expert radiologists (R.E.Y. and S.H.C. with 16 and 23 years of experience in neuroradiology, respectively) and were applied uniformly across all subjects.

### 2.6 PVS identification and quantification

The initial PVS mask was multiplied with WM and BG masks obtained from preprocessing steps to eliminate false positives (FP) in non-tissue regions. Each connected component from the resulting segmentation was individually considered as potential PVS regions for further FP reduction. To address the occurrence of some FPs along the periventricular WMH regions, a dilated lateral ventricle mask was created to exclude any candidates that overlapped in this region. The remaining candidates were then further scrutinized for FP reduction based on criteria of size and linearity. For size, a minimum voxel count threshold of 3 voxels was applied to eliminate small noise fragment FPs amongst the candidates.

To exclude candidate regions with blob-like rather than tubular morphology, we applied a linearity filter based on each candidate’s shape characteristics. Specifically, for every connected component, we computed (i) its longest axis length in voxels and (ii) its total area in voxels. Candidates were retained only when (longest axis length × an empirically determined weight) was greater than or equal to the total area, which preserved elongated tubular shapes characteristic of true perivascular spaces while excluding approximately round or irregular candidates — typically corresponding to lacunar infarcts, DWMH, or other hyperintense vascular pathology that survive the prior FP reduction steps [47]. Weights for linearity determination were optimized separately for candidates in WM and BG, as appearance of PVS in BG is more lenient to non-linear shapes [15]. These weights were optimized through visual inspection of the two expert radiologists.

Resulting PVS candidates were masked into the four lobar WM and BG regions for subsequent analysis. Regional PVS burden were calculated by voxel counts of remaining segmented candidates in each region, and normalized by intracranial volumes (ICV) [29]. An overview of the segmentation pipeline is outlined in Fig. 1. We then compared the quantified PVS volumes with conventional visual ratings by scoring enlarged PVS on axial T2-weighted images using a validated 5-point scale [15]. Representative slices with the highest PVS burden in the BG and CSO were selected, and PVS were counted regardless of their size using the following grades: 0 (0 PVS), 1 (1–10), 2 (11–20), 3 (21–40), and 4 (>40). In cases of hemispheric asymmetry, the higher rating was recorded.

### 2.7 Vascular burden assessment and amyloid-β positivity determination

The two experienced radiologists assessed the presence or absence of signs of SVD on conventional MRI by consensus. The signs of SVD included both periventricular and deep WMH (PVWMH, DWMH), lacunes, prominent PVS, and CMB [1]. PVWMH and DWMH were visually graded as 0-1 (mild), 2 (moderate), or 3 (severe) using the Fazekas scale [51]. CMBs were evaluated on SWI and defined as small (generally 2–5 mm in diameter, but up to 10 mm) areas of signal void with associated blooming [52]. Participants were classified as vascular burden positive (VB+) if any of the following were present: (1) more than a few CMBs, (2) any lacune, (3) PVWMH Fazekas score ≥ 2 or DWMH Fazekas score ≥ 2; otherwise, they were classified as vascular burden negative (VB-). Participants were further stratified by pathology severity using CMB counts (0, 1–4, ≥5) [53], lacune presence (yes/no), PVWMH Fazekas scale (0–1, 2, 3), and DWMH Fazekas scale (0–1, 2, 3).

The presence of Aβ pathology was defined using global SUVR values. A two-component Gaussian mixture model (GMM) was fitted to global SUVR values across all 307 participants to derive a data-driven cutoff by estimating the posterior probability of belonging to the high-SUVR cluster. The cutoff was defined at a posterior probability of 0.5 and was estimated at 1.25; participants with global SUVR above the cutoff were classified as Aβ burden positive (AB+), and the rest as Aβ burden negative (AB−). For severity analyses, FBB PET images at various brain levels—including the frontal, cingulate, parietal, temporal, and cerebellar cortices—were visually assessed using the Brain Amyloid-β Plaque Load (BAPL) scoring system (1: no Aβ load, 2: minor Aβ load, 3: significant Aβ load) [54].

### 2.8 Statistical analysis

Comparisons of demographics and imaging biomarkers were assessed using chi-square tests for categorical variables, and Kruskal-Wallis test with Dunn’s post hoc pairwise analysis for continuous variables. Regional PVS were log-transformed for all statistical analyses to reduce right-skewness and to better satisfy model assumptions for analyses of covariance (ANCOVA) and multivariable linear regression (MLR).

To test whether pathology-associated patterns of PVS burden differed across brain regions, participants were regrouped into four pathology groups based on VB and AB status: pathology-negative (VB− AB−), single VB-positive (VB+ AB−), single AB-positive (VB− AB+), and double-positive (VB+ AB+). Regional log-transformed PVS was compared between pathology groups using ANCOVA adjusted for age and sex. For each region, the four p-values were corrected using the Benjamini–Hochberg false discovery rate (FDR) procedure.

Next, we evaluated whether regional PVS differed across increasing pathology severity defined by Aβ markers (global SUVR and BAPL) and vascular markers (CMB, lacunes, PVWMH, and DWMH). To minimize confounding between Aβ and vascular pathology, analyses of Aβ severity were performed in VB- participants, and analyses of vascular severity were performed in AB- participants. For each marker, groupwise and post-hoc pairwise comparisons of regional log‑transformed PVS across severity subgroups were performed using ANCOVA, adjusting for age and sex in VB− participants and for age, sex, and non‑predictor vascular covariates in AB− participants. Effect sizes are reported as partial η² for groupwise and Cohen’s d for pairwise comparisons. P-values were corrected across regions using the FDR procedure for each set of comparisons. In VB- participants, associations between global Aβ SUVR and regional log-transformed PVS were additionally assessed using Pearson (linear) and Spearman (monotonic) correlations. Then, to assess Aβ-vascular interaction effects, we fit MLR models with regional log-transformed PVS as the outcome and AB positivity, VB positivity, and their interaction term (AB×VB) as predictors, adjusting for age and sex. We then examined marker‑specific interactions by replacing VB with binary vascular markers and the corresponding interaction terms (AB×CMB, AB×lacunes, and AB×WMH). CMB status was defined as any versus none, lacune status as present versus absent, and WMH status as positive if PVWMH Fazekas ≥ 2 or DWMH Fazekas ≥ 2. Vascular marker–by–vascular marker interactions were not included because additive effects were assumed. For each model, p-values were corrected across regions using the FDR procedure for each predictor.

## 3 Results

### 3.1 Demographics and clinical characteristics of study participants

Out of a total of 307 cognitively impaired participants included in this study, 36 were AB- VB-, 106 were AB- VB+, 48 were AB+ VB-, and 117 were AB+ VB+ (Table 1). Sex and years of education did not vary significantly amongst the four groups, while age was significantly higher in all VB+ groups as compared to all VB- groups (Kruskal-Wallis: *H*=52.0, FDR-corrected *p*<0.001). All AB+ groups showed significantly lower MMSE-KC scores than all AB- groups (Kruskal-Wallis: *H*=18.5, FDR-corrected *p*<0.001), while CDR scores only differed significantly between AB- VB+ and AB+ VB- groups (Kruskal-Wallis: *H*=10.3, FDR-corrected *p*=0.02).

**Table 1:** Demographics and imaging measures of study cohort.

		AB- VB-	AB- VB+	AB+ VB-	AB+ VB+	p-value
Subjects (*n*)		36	106	48	117	
Age (years)		70.0 ± 6.98	77.4 ± 6.48	70.3 ± 9.80	78.1 ± 5.30	<0.001^a,c,d,f^
Sex (Male/Female)		12/24	41/65	19/29	43/74	0.93
Education (years)		9.94 ± 4.42	10.6 ± 4.96	11.4 ± 4.58	10.4 ± 5.04	0.47
MMSE-KC		23.5 ± 4.56	23.3 ± 3.99	20.8 ± 5.15	21.0 ± 4.86	<0.001^b-e^
CDR		0.92 ± 0.94	1.03 ± 1.46	1.64 ± 2.31	1.23 ± 1.69	0.02 ^d^
Global Amyloid-β SUVR		1.05 ± 0.09	1.07 ± 0.07	2.17 ± 0.44	2.06 ± 0.48	<0.001^b-e^
BAPL Score (*n*)	1	34	102	1	16	<0.001^b^, ^c^, ^d^, ^e^
	2	2	3	8	20	
	3	0	1	39	81	
DWMH Fazekas Score (*n*)	0	3	1	2	2	<0.001^a^^,^^c^^,^^d^^,^^f^
	1	33	57	46	61	
	2	0	40	0	50	
	3	0	8	0	4	
PVWMH Fazekas Score (*n*)	0	0	0	3	0	<0.001^a^^,^^c^^,^^d^^,^^f^
	1	36	14	45	13	
	2	0	48	0	65	
	3	0	44	0	39	
CMB Counts (*n*)	0	23	57	35	55	0.02
	1-4	13	34	13	50	
	5≤	0	15	0	12	
Presence of Lacunes (*n*)		0	29	0	36	<0.001^a,c,d,f^

Data are presented as mean ± standard deviation for continuous variables and number for nominal variables.

Independent Kruskal-Wallis test with Dunn’s post-hoc pairwise comparisons for continuous variables and chi-square test for nominal variables.

Abbreviations: AB = Amyloid-β Burden, VB = Vascular Burden, MMSE-KC = Mini-Mental State Examination in the Korean Version of the CERAD Assessment Packet, CDR = Clinical Dementia Rating, SUVR = Standardized Uptake Value Ratio, BAPL = Brain Amyloid-β Plaque Load, DWMH = Deep White Matter Hyperintensity, PVWMH = Periventricular White Matter Hyperintensity, CMB = Cerebral Microbleed.

a Significant differences (p<0.05) between AB- VB- and AB- VB+ groups

b Significant differences (p<0.05) between AB- VB- and AB+ VB- groups

c Significant differences (p<0.05) between AB- VB- and AB+ VB+ groups

d Significant differences (p<0.05) between AB- VB+ and AB+ VB- groups

e Significant differences (p<0.05) between AB- VB+ and AB+ VB+ groups

f Significant differences (p<0.05) between AB+ VB- and AB+ VB+ groups

All VB− groups had Fazekas scores of 0–1 for both DWMH and PVWMH, resulting in significantly lower WMH severity than all VB+ groups (Chi-Square: *χ^2^*=64.0/216.6, FDR-corrected *p*<0.001/0.001, DWMH/PVWMH). Presence of lacunes was also non-existent in all VB- groups and thus were significantly varied compared to all VB+ groups (Chi-Square: *χ^2^*=31.4, FDR-corrected *p*<0.001). CMB severity by count showed significant difference across the four groups (Chi-Square: *χ^2^*=15.6, FDR-corrected *p*=0.02) but did not reveal any significant differences between individual group pairs. All AB- groups had significantly lower global Aβ SUVR and BAPL scores compared to AB+ groups (Kruskal-Wallis: *H*=229.3, FDR-corrected *p*<0.001).

### 3.2 Reproducibility of automated PVS quantification

We compared automated PVS quantification with visual PVS ratings to validate whether the automated tool captures the underlying PVS distribution. In the BG, participants were distributed across rating grades as follows: grade 0 (*n*=0), grade 1 (*n*=100), grade 2 (*n*=147), grade 3 (*n*=57), and grade 4 (*n*=3). In the CSO, the distribution was grade 0 (*n*=0), grade 1 (*n*=35), grade 2 (*n*=123), grade 3 (*n*=119), and grade 4 (*n*=30). The proportion of high BG burden (grade≥2) differed significantly across AB/VB subgroups (*p*<0.001), whereas CSO grades did not (*p*=0.09). Automated measures showed moderate agreement with visual ratings in both regions, with Spearman correlations of *ρ*=0.589 for BG (*p*<0.001) and *ρ*=0.559 for CSO (*p*<0.001). Visual BG grades were compared against automated BG PVS burden, and visual CSO grades were compared against automated frontoparietal PVS burden.

### 3.3 Distinct spatial patterns of PVS burden across multiple pathologies

**Fig. 2:**
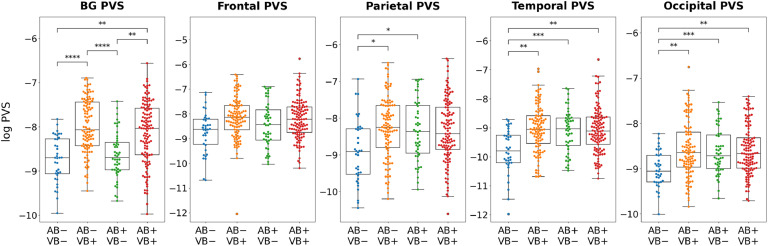
Pairwise Comparisons of PVS Between Pathological Groups by Region. Regional log-transformed PVS boxplots across five regions for AB/VB positivity subgroups (AB- VB-, AB- VB+, AB+ VB-, AB+ VB+). Pairwise group differences were tested using ANCOVA with FDR correction per region. Only significant corrected p-values are shown. Significance: * p<0.05, ** p<0.01, *** p<0.001, **** p<0.0001.

We then examined whether pathology-associated patterns of PVS burden differed across brain regions. Regional PVS burden differed significantly across the four pathology groups in all regions except the frontal lobe (partial *η²*=0.04–0.12, all FDR-corrected *p*≤0.013; Supplementary Table 2). As illustrated in Table 2 and Fig. 2, the parietal, temporal, and occipital lobes showed a similar pattern in terms of which pathologies had elevated PVS, which was distinct from that observed in the BG and frontal regions. In the BG, VB positivity was associated with significantly higher PVS regardless of AB status.

**Table 2:** Statistic values of pairwise comparisons of regional log-transformed PVS between pathological subgroups.

Parameter	BG PVS	Frontal PVS	Parietal PVS	Temporal PVS	Occipital PVS
Comparison between pathology-negative (AB− VB−) and single vascular burden (AB− VB+)					
F	26.97	3.62	8.25	12.48	9.70
p-value	<0.0001****	0.36	0.014*	0.002**	0.004**
Cohen's d (95% CI)	1.28 (0.87, 1.68)	0.69 (0.30, 1.07)	0.77 (0.38, 1.16)	0.98 (0.59, 1.38)	0.77 (0.38, 1.16)
Comparison between pathology-negative (AB− VB−) and single amyloid-β burden (AB+ VB−)					
F	0.47	2.48	9.33	18.04	18.03
p-value	0.49	0.36	0.014*	<0.001***	<0.001***
Cohen's d (95% CI)	0.17 (−0.26, 0.60)	0.33 (−0.10, 0.77)	0.66 (0.22, 1.11)	0.93 (0.47, 1.38)	0.86 (0.41, 1.32)
Comparison between pathology-negative (AB− VB−) and mixed-pathology group (AB+ VB+)					
F	10.19	1.54	2.94	8.77	11.61
p-value	0.003**	0.43	0.18	0.007**	0.002**
Cohen's d (95% CI)	0.91 (0.52, 1.30)	0.65 (0.27, 1.03)	0.66 (0.28, 1.04)	0.94 (0.55, 1.33)	0.84 (0.46, 1.23)
Comparison between single vascular (AB− VB+) and single amyloid burden (AB+ VB−)					
F	29.13	0.12	0.008	0.13	0.34
p-value	<0.0001****	0.87	0.93	0.72	0.68
Cohen's d (95% CI)	−1.13 (−1.49, −0.76)	−0.33 (−0.68, 0.01)	−0.10 (−0.45, 0.24)	−0.10 (−0.44, 0.24)	−0.08 (−0.42, 0.26)
Comparison between single vascular (AB− VB+) and mixed-pathology group (AB+ VB+)					
F	3.07	0.46	0.65	0.29	0.05
p-value	0.10	0.75	0.51	0.72	0.82
Cohen's d (95% CI)	−0.22 (−0.48, 0.05)	−0.06 (−0.33, 0.20)	−0.10 (−0.36, 0.16)	−0.05 (−0.32, 0.21)	−0.03 (−0.29, 0.24)
Comparison between single amyloid burden (AB+ VB−) and mixed-pathology group (AB+ VB+)					
F	12.34	0.02	0.81	0.44	0.50
p-value	0.001**	0.90	0.51	0.72	0.68
Cohen's d (95% CI)	0.78 (0.44, 1.13)	0.28 (−0.05, 0.62)	0.00 (−0.33, 0.34)	0.05 (−0.29, 0.38)	0.06 (−0.28, 0.40)

All PVS volumes are log-transformed.

All reported p-values are corrected using the Benjamini-Hochberg FDR procedure.

FDR-corrected significance: *p<0.05, **p<0.01, ***p<0.001, ****p<0.0001.

Abbreviations: BG = Basal Ganglia, PVS = Perivascular Spaces, AB = Amyloid-β Burden, VB = Vascular Burden, FDR = False Discovery Rate, CI = Confidence Interval.

In the parietal, temporal, and occipital lobes, both VB and AB were associated with higher PVS, but we found no evidence that co-existence of VB and AB was accompanied by a further increase in PVS beyond either pathology alone. Relative to the pathology-negative group (AB- VB-), both single-pathology groups (AB- VB+ and AB+ VB-) showed significantly higher PVS across these regions, whereas no significant differences were observed between either the single pathology group and the mixed-pathology group (AB+ VB+) (FDR-corrected *p*>0.1 for all comparisons across all three regions).

### 3.4 Association between pathology severity and regional PVS burden

**Fig. 3:**
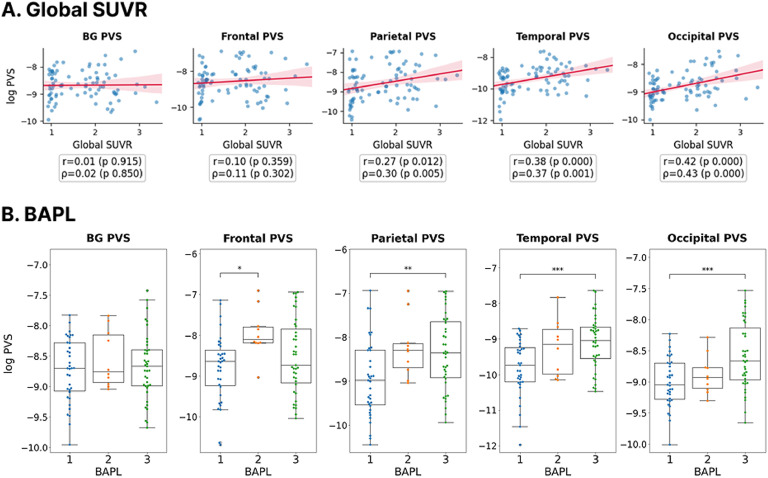
Associations Between Amyloid-β Severity and Regional PVS in VB− Participants. (A) Scatterplots for each region showing global SUVR (x-axis) versus log-transformed PVS (y-axis) in VB− participants. The fitted line represents the linear regression. Pearson’s r and Spearman’s ρ are reported with p-values. (B) Regional log-transformed PVS boxplots across five regions by BAPL grade. Pairwise comparisons were performed using ANCOVA adjusted for age and sex. P-values were corrected within each region using the FDR procedure; only significant corrected p-values are shown. Significance: * p<0.05, ** p<0.01, *** p<0.001, **** p<0.0001.

We observed varied increases in regional PVS burden with greater pathological marker severities. First, we evaluated Aβ severity relations to regional PVS in VB- groups (*n*=84). Higher Aβ retentions were significantly related to larger PVS in the parietal, temporal, and occipital lobes. Global SUVR showed significantly positive correlations with log-transformed PVS in each of these regions (Fig. 3.A). A corresponding pattern was also observed in a pairwise comparison of PVS between BAPL severity subgroups, with BAPL 3 subgroups showing significantly higher parietal, temporal, and occipital PVS as compared to BAPL 1 subgroups (Fig. 3.B). However, the increase in PVS did not appear strictly linear in this analysis, as no significant increase was observed between the BAPL 2 subgroup as compared to the other two subgroups.

**Fig. 4:**
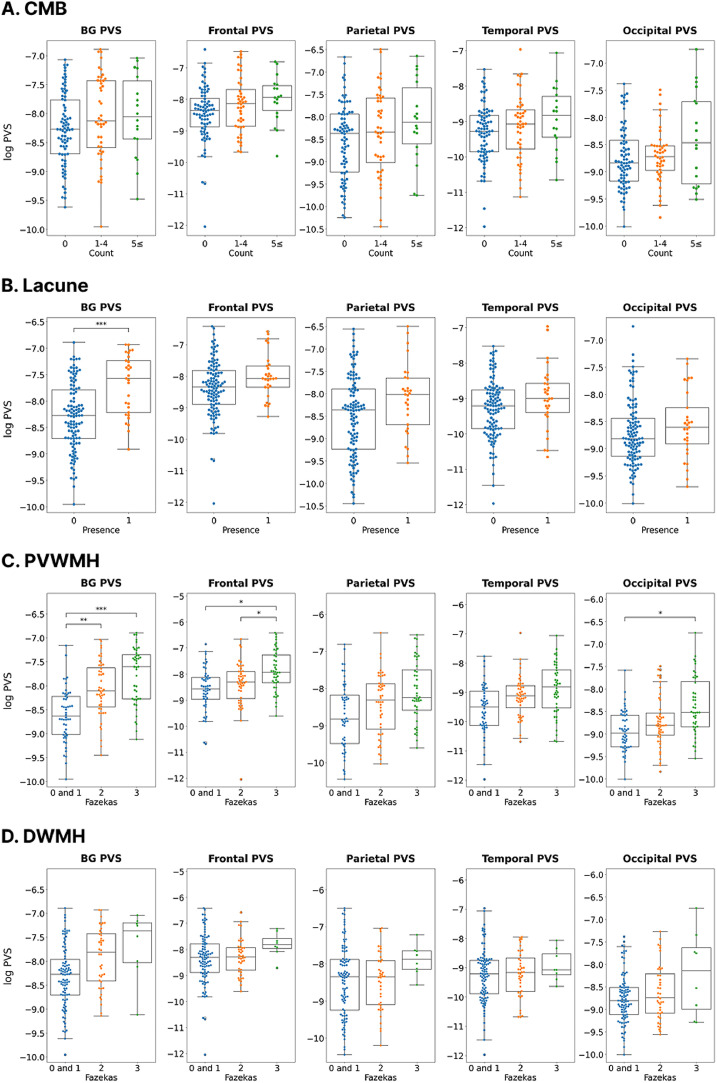
Associations Between Vascular Burden Severity and Regional PVS in AB− Participants. Regional log-transformed PVS boxplots across five regions, stratified by (A) CMB count, (B) lacune presence, (C) PVWMH Fazekas scale, and (D) DWMH Fazekas scale. Pairwise comparisons were performed using ANCOVA with covariate adjustment as follows: (A) age, sex, lacune, PVWMH, and DWMH; (B) age, sex, CMB counts, PVWMH, and DWMH; (C) age, sex, CMB counts, lacune, and DWMH; and (D) age, sex, CMB counts, lacune, and PVWMH. All p-values were corrected within each region (per marker) using the FDR procedure; only significant corrected p-values are shown. Significance: * p<0.05, ** p<0.01, *** p<0.001, **** p<0.0001.

We further investigated regional PVS patterns in increasing severities of vascular pathology related markers within AB- subjects (*n*=142) (Fig. 4). Among these markers, PVWMH severity demonstrated the strongest association with PVS burden, with progressive increase in PVS observed across increasing Fazekas grades in the BG, frontal, and occipital regions (Fig. 4.C). Among these regions, the BG showed significant differences between Fazekas 0 and 1 subgroup as compared to Fazekas 3 subgroup. The BG region also solely expressed significant increase in PVS in the presence of lacunes (Fig. 4.B). Although elevated trends of PVS with increasing severity of CMB burden and DWMH Fazekas ratings were observed, these increases did not show statistical significance at any region (Fig. 4.A and Fig. 4.D).

### 3.5 Distinct spatial patterns of PVS burden for Co-existing pathologies

**Fig. 5:**
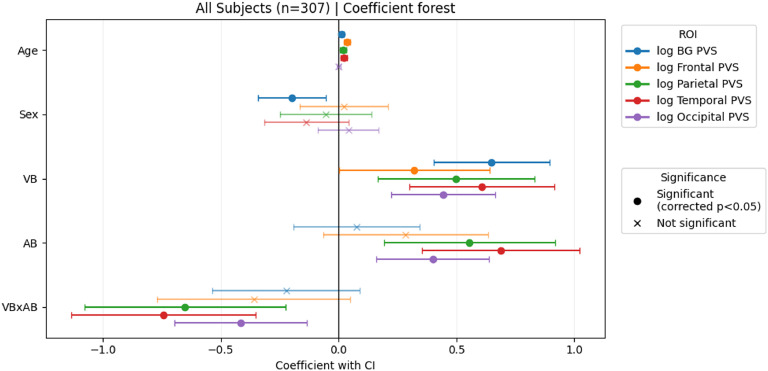
Regional Amyloid-Vascular Interaction Effects on PVS. Forest plot of multivariable linear regression coefficients and 95% confidence intervals (CI) for models predicting regional log-transformed PVS from amyloid-β burden (AB) status, vascular burden (VB) status, and their interaction (AB×VB), adjusted for age and sex. Coefficients and CIs are color-coded by region. For each predictor, p-values were corrected across regions using the FDR procedure. Circles with thick CI bars indicate FDR-significant effects (corrected p<0.05); crosses with thin CI bars indicate non-significant effects.

We fit MLR interaction models to test whether the associations of AB and VB positivity with regional PVS burden were mutually modifying. In the parietal, temporal, and occipital lobes, the AB main effect, VB main effect, and AB×VB interaction were all statistically significant. (AB *β*=0.40–0.69, VB *β*=0.44–0.61, AB×VB *β*=−0.42 to −0.74; all FDR-corrected *p*≤0.006) The negative interaction coefficients indicate that the joint association of AB and VB with regional PVS volume is less than would be expected if their individual effects were combined additively. As shown by the marginal-mean estimates in Supplementary Table 3, PVS volumes in the mixed-pathology group are comparable to or only marginally lower than those in the single-pathology groups, with overlapping confidence intervals. (Fig. 5, Table 3) In this model, the AB main effect reflects the AB+ vs AB− difference in PVS among VB− participants, whereas the VB main effect reflects the VB+ vs VB− difference among AB− participants; both effects were significant in these regions. Follow-up conditional effect analyses further showed that AB status was not significantly associated with PVS among VB+ participants, and VB status was not significantly associated with PVS among AB+ participants (Supplementary Table 4).

**Table 3:** Multivariable linear regression of amyloid-vascular effects on regional PVS volume.

Parameter	BG PVS	Frontal PVS	Parietal PVS	Temporal PVS	Occipital PVS
VB					
β (95% CI)	0.65 (0.40, 0.89)	0.32 (0.00, 0.64)	0.50 (0.17, 0.83)	0.61 (0.30, 0.91)	0.44 (0.22, 0.66)
p-value	<0.0001****	0.049*	0.004**	<0.001***	<0.001***
AB					
β (95% CI)	0.08 (−0.19, 0.34)	0.28 (−0.06, 0.63)	0.55 (0.19, 0.92)	0.69 (0.35, 1.02)	0.40 (0.16, 0.64)
p-value	0.58	0.14	0.005**	<0.001***	0.003**
VB×AB					
β (95% CI)	−0.22 (−0.53, 0.09)	−0.36 (−0.77, 0.05)	−0.65 (−1.08, −0.23)	−0.74 (−1.13, −0.35)	−0.42 (−0.70, −0.14)
p-value	0.16	0.10	0.006**	0.001**	0.006**

All PVS volumes are log-transformed.

All reported p-values are corrected using the Benjamini-Hochberg FDR procedure.

FDR-corrected significance: *p<0.05, **p<0.01, ***p<0.001, ****p<0.0001.

Abbreviations: BG = Basal Ganglia, PVS = Perivascular Spaces, AB = Amyloid-β Burden, VB = Vascular Burden, FDR = False Discovery Rate, CI = Confidence Interval.

By contrast, in the BG and frontal regions, only the VB main effect remained statistically significant, with no evidence of either independent AB effect or an AB×VB interaction. This regional pattern indicates that variations in PVS in the BG and frontal lobe are primarily driven by VB positivity and are not significantly modified by AB status (Fig. 5).

We further assessed marker-specific amyloid-vascular interaction in an MLR model incorporating AB positivity, individual vascular markers, and their corresponding interaction terms. Similarly to the previous MLR results, the temporal and occipital lobes showed significant main effects of AB and WMH along with a significant AB×WMH interaction. However, the parietal lobe showed only a significant effect of AB status, with neither main effects of vascular markers nor their interactions with AB (Supplementary Fig. 2, Supplementary Table 5).

## 4 Discussion

In this study, we investigated how Aβ and vascular driven pathologies relate to anatomical distributions of enlarged PVS, and whether their pathological markers interact across distinct brain regions. Overall, our findings demonstrate pronounced regional heterogeneity in pathology-associated PVS burden, with BG and lobar WM regions exhibiting different spatial patterns of vulnerability. Specifically, PVS burden within the parietal, temporal, and occipital lobes was consistently associated with AB, whereas BG PVS burden was predominantly linked to VB. Such spatially divergent patterns suggest multiple underlying mechanisms of regional glymphatic related alterations in AD and VD, rather than a global pathological process. Furthermore, our findings demonstrated that the contribution of the two pathologies to regional PVS burden was less-than-additive – a statistical pattern that admits several potential biological interpretations.

We observed that the parietal, temporal, and occipital lobes exhibited highly similar patterns of PVS dilation that were affected by both AB and VB positivity, whereas BG showed PVS burden almost exclusively driven by VB. This regional dissociation could potentially highlight the differences in the biological processes that govern glymphatic alterations in lobar and deep regions. Lobar PVS burden may primarily reflect perivascular dilation due to disruption of clearance pathways that are sensitive to both Aβ deposition and vascular integrity [13,29]. In contrast, BG conduciveness to PVS burden may be more likely to represent structural consequences of SVD, including arteriolosclerosis and ischemic injury affecting deep perforating arteries [47].

In addition, the preferential involvement of temporal and occipital regions of AB driven PVS burden is particularly notable, as these areas are well recognized as primary sites of vulnerability in the AD continuum. AD related neurodegenerative processes characteristically involve medial temporal structures that subsequently extend to posterior cortical regions, including lateral temporal and occipital areas [55]. The spatial concordance between these vulnerable regions and AB associated PVS burden observed in our study is consistent with previously reported anatomical patterns of AD-associated glymphatic-related alterations [6].

In contrast to lobar regions, BG PVS burden was strongly associated with vascular pathology markers, particularly PVWMH severity and lacune presence, and showed no significant evidence of modulation by AB status. This finding is consistent with prior literature identifying BG PVS as a hallmark of cerebral SVD, closely linked with impaired perforating artery function [7,8]. The absence of AB-VB interactions in BG region further supports the interpretation that BG PVS burden is primarily driven by VB, and this relation is not modified by AB.

Our MLR model further supported these subgroup findings, showing significant independent effects of both AB and VB on regional PVS burden. VB was independently associated with PVS burden across all regions, whereas AB was independently associated with parietal, temporal, and occipital regions. However, the negative AB×VB interaction term indicated a less-than-additive effect: when both pathologies co-existed, PVS burden did not increase beyond the level attributable to a single pathology. The less-than-additive AB×VB interaction observed in the lobar regions admits multiple biological interpretations that cannot be uniquely distinguished by the present cross-sectional data. One possibility is a saturable structural mechanism, in which physical, biological, or imaging-resolution constraints on perivascular dilation impose a ceiling: once a single dominant pathology has sufficiently expanded the measurable PVS compartment, additional pathological burden may confer diminishing marginal effects on observable PVS volume. An equally plausible alternative is that mixed-pathology participants who present clinically at this stage represent a selected subset in which underlying PVS trajectories differ from those of single-pathology participants in ways that could mimic a saturation pattern without reflecting a true biological ceiling. Distinguishing among these accounts will require longitudinal imaging, alternative modalities sensitive to functional glymphatic activity, and cohorts spanning a broader range of disease severity [21].

The effects of the interaction between AB and VB positivity were analyzed more deeply through our MLR model that incorporated vascular markers along with their interactions to AB status. In the temporal and occipital lobes, both AB and WMH showed significant main effects, accompanied by a less-than-additive AB×WMH interaction. Given that WMH frequently co-occur with AD and preferentially affect posterior regions, the observed main effects and less-than-additive AB×WMH interaction are reasonable. In other words, where one pathology had already elevated PVS burden, the additional contribution of the other was modest – the same less-than-additive pattern seen in the primary AB×VB interaction analysis [56]. In contrast, WMH and presence of lacunes showed significant main effects on BG PVS, with no significant interaction effects with AB. Taken together, our findings consistently showed predominant vulnerability of BG PVS to vascular factors such as PVWMH and lacunes, which supports the previously reported association of BG PVS burden with hypertensive arteriopathy and other markers of SVD [[8], [9], [10]]. Interestingly, the parietal lobe demonstrated significant AB×VB interaction when vascular burden was modeled globally, yet no significant interactions emerged when individual vascular markers were examined. This discrepancy suggests that parietal PVS burden may be sensitive to diffuse vascular dysfunction rather than to specific lesion types such as WMH, lacunes, or CMBs [57]. Finally, the frontal lobe showed no significant predictor in this comprehensive model, whereas the simpler AB, VB status model revealed a marginal VB effect.

The relative absence of an AB association with frontal-lobe PVS, despite well-established early frontal parenchymal Aβ deposition along the Thal staging continuum, may be partially explained by the dissociation between parenchymal and vascular amyloid topography. PVS burden may more closely reflect vascular Aβ deposition in the form of CAA than parenchymal Aβ plaques, and CAA characteristically affects posterior vasculature — occipital and parietal regions — preferentially. The marginal VB association observed in the frontal lobe (*β*=0.32; FDR-corrected *p*=0.049), on the other hand, suggests that frontal PVS may be driven primarily by SVD rather than by an AD-specific process. This interpretation is also consistent with our broader regional pattern: the posterior-predominant amyloid-related PVS signal observed across the temporal, parietal, and occipital lobes is anatomically congruent with the established posterior predominance of CAA [7,8].

Our quantification of PVS represents a structural and indirect surrogate of glymphatic system status rather than a direct measure. We did not directly measure dynamic glymphatic flow, clearance kinetics, or CSF–interstitial fluid exchange. Moreover, enlarged PVS are not specific to glymphatic dysfunction. Brain atrophy, neuroinflammation, and blood–brain barrier dysfunction may also contribute independently to PVS dilation, and our cross-sectional design cannot isolate the relative contributions of these mechanisms [[58], [59], [60]]. Nonetheless, enlarged PVS may still partly reflect glymphatic status, and once enlarged, they may further compromise glymphatic transport through several physiological consequences. First, the enlarged perivascular lumen may attenuate the efficiency with which arterial wall motion — driving force for glymphatic flow — is transmitted to the surrounding fluid, dampening pulsatile CSF propulsion along the periarterial compartment [61,62]. Second, the expanded compartment may reduce the directional bulk-flow efficiency along these channels [63]. Third, the increased physical distance between the periarterial compartment and adjacent parenchyma may reduce the efficiency of CSF–interstitial fluid exchange. Together, these effects suggest a potential vicious-cycle dynamic in which enlarged PVS are not merely a passive marker of glymphatic dysfunction but may also actively contribute to its progression.

Several considerations should be noted when interpreting our findings. First, the pathology-negative AB-VB- reference subgroup was relatively smaller (*n*=36) than the AB- VB+ (*n*=106) and AB+ VB+ (*n*=117) subgroups, limiting statistical power for detecting modest effects, particularly in the AB+ VB- subgroup (*n*=48) and in regions with smaller mean differences such as the frontal lobe. This imbalance partly reflects the source population, as VB pathology is common and accrues with age in clinical dementia cohorts. Second, our Aβ positivity threshold (global SUVR>1.25) was derived from a two-component GMM fitted to the same cohort. Although this data-driven approach accommodates cohort- and pipeline-specific differences in PET intensity scaling, it has not been externally validated and may not generalize to other cohorts. However, a continuous SUVR sensitivity analysis (Supplementary Table 6) reproduced the regional pattern of AB-related PVS burden across all five regions, indicating that our conclusions are independent of the specific threshold. Third, the agreement between our automated PVS volumes and conventional manual visual ratings was moderate, likely reflecting the differing nature of the two approaches — categorical rating captures gross severity within prespecified slices, whereas automated quantification captures finer regional variation. We therefore interpret the automated measure as complementary to, rather than a replacement for, visual assessment.

Beyond these, several aspects warrant consideration in future work. First, the cross-sectional design precludes inference regarding temporal causality between pathological and PVS burden. Longitudinal imaging will be essential to determine whether regional PVS changes precede, accompany, or follow AB- and VB-driven pathology. Second, although we examined multiple vascular markers, these may not fully capture diffuse vascular dysfunction such as endothelial impairment, arterial stiffness, or altered cerebrovascular pulsatility, which may be particularly relevant to glymphatic transport. Third, our analysis did not incorporate other neurodegenerative pathologies associated with cognitive impairment, such as Parkinson’s disease or Lewy body pathology, which may also involve glymphatic dysfunction and distinct PVS patterns. Incorporating physiological vascular metrics, dynamic imaging, and broader disease spectra in future work will help clarify the mechanisms and generalizability of the observed regional PVS signatures.

## 5 Conclusion

In summary, this study demonstrates that PVS burden exhibits marked spatial patterns in its association with Aβ and vascular pathologies, reflecting anatomically distinct mechanisms rather than a global pathological response. These findings underscore the importance of spatially informed interpretation of glymphatic system biomarkers and suggest that regional patterns of PVS burden may serve as a sensitive, quantifiable neuroimaging marker for distinguishing mixed pathological processes. Future longitudinal and multimodal investigations will be essential to clarify the temporal dynamics and mechanistic significance of PVS accumulation and enlargement, and to establish its utility for accurate cognitive impairment stratification.

## Ethics and consent statement

This retrospective cohort study was approved by the institutional review board of the Seoul National University Hospital, with the requirement for informed consent waived (IRB No. 2311-048-1482). The study protocol was performed in accordance with the Declaration of Helsinki.

## Declaration of the use of generative AI and AI-assisted technologies in scientific writing and in figures, images and artwork

During the preparation of this work the authors did not use any generative AI or AI-assisted technologies in the creation of all the contents in this manuscript.

## Fundings

This work was supported by the National Research Foundation of Korea (NRF) grant funded by the Korea government (MSIT) (NRF-2023R1A2C3003250, R.E.Y.). This study was supported by a grant of the Korea Health Technology R&D Project through the Korea Health Industry Development Institute (KHIDI), funded by the Ministry of Health & Welfare, Republic of Korea (RS-2023-00262321, R.E.Y.). This work was supported by the National Research Foundation of Korea (NRF) grant funded by the Korea government (MSIT) (IRIS RS-2026-25488643). This work was supported by a grant of the Korea Health Technology R&D Project through the Korea Health Industry Development Institute (KHIDI), funded by the Ministry of Health & Welfare, Republic of Korea (RS-2025-02307233). This work was financially supported by the Ministry of Small and Medium-sized Enterprises (SMEs) and Startups (MSS), Korea, under the “SME Technology Innovation Development Program (R&D, RS-2025-25450379)” supervised by the Korea Technology and Information Promotion Agency for SMEs. This work was financially supported by “Seoul R&BD Program (BT240161)”, supervised by the Seoul Business Agency.

## CRediT authorship contribution statement

**Woosik Kim:** Writing – original draft, Visualization, Software, Methodology, Investigation, Data curation, Conceptualization. **Yejin Hwang:** Writing – original draft, Validation, Investigation, Data curation. **Yelim Yang:** Writing – original draft, Validation, Investigation, Data curation. **Min Gyeong Kim:** Visualization, Investigation, Formal analysis. **Hyemin Jang:** Writing – review & editing, Methodology. **Seung Hong Choi:** Resources, Data curation. **Joon-Kyung Seong:** Writing – review & editing, Resources, Project administration, Conceptualization. **Roh-Eul Yoo:** Writing – review & editing, Supervision, Project administration, Data curation, Conceptualization. **Wha Jin Lee:** Writing – review & editing, Supervision, Resources, Project administration, Conceptualization.

## Declaration of competing interest

The authors declare the following financial interests/personal relationships which may be considered as potential competing interests:

Woosik Kim reports a relationship with NeuroXT Inc. that includes: employment. Min Gyeong Kim reports a relationship with NeuroXT Inc. that includes: employment. Joon-Kyung Seong reports a relationship with NeuroXT Inc. that includes: equity or stocks. Roh-Eul Yoo reports a relationship with NeuroXT Inc. that includes: consulting or advisory. Wha Jin Lee reports a relationship with NeuroXT Inc. that includes: employment and equity or stocks. If there are other authors, they declare that they have no known competing financial interests or personal relationships that could have appeared to influence the work reported in this paper.

## Data Availability

The dataset and pipeline used and/or analysed during the current study are available from the corresponding authors on reasonable request.

## Appendix Supplementary materials

Image, application 1
