# Supplementary material for: Distinct spatial patterns of perivascular spaces enlargement for multiple and Co-existing pathologies of cognitive impairment
Source: J Prev Alzheimers Dis. 2026 Jul 1;13(8):100631. doi: 10.1016/j.tjpad.2026.100631 (PMC13355189; doi:10.1016/j.tjpad.2026.100631)
Supplement: Supplementary file 1 [file mmc1.docx]

Supplementary Materials

Distinct Spatial Patterns of Perivascular Spaces Enlargement for Multiple and Co-existing Pathologies of Cognitive Impairment

Woosik Kim^1,2†^, Yejin Hwang^3†^, Yelim Yang^3†^, Min Gyeong Kim^2^, Hyemin Jang^4^, Seung Hong Choi^3,5^, Joon-Kyung Seong^1,2,6^, Roh-Eul Yoo^3,5*^ and Wha Jin Lee^2*^

**^†^These authors contributed equally to this work.**

**^*^Co-corresponding Authors**

^1^School of Biomedical Engineering, Korea University, 145, Anam-ro, Seongbuk-gu, Seoul, Republic of Korea

^2^AI Imaging Research Center, NeuroXT, Inc., 48, Achasan-ro 17-gil, Seongdong-gu, Seoul, Republic of Korea

^3^Department of Radiology, Seoul National University College of Medicine, 101, Daehangno, Jongno-gu, Seoul, Republic of Korea

^4^Department of Neurology, Asan Medical Center, University of Ulsan College of Medicine, 88, Olympic-ro 43-gil, Songpa-gu, Seoul, Republic of Korea

^5^Department of Radiology, Seoul National University Hospital, 101, Daehangno, Jongno-gu, Seoul, Republic of Korea

^6^Department of Artificial Intelligence, Korea University, 145, Anam-ro, Seongbuk-gu, Seoul, Republic of Korea

# Supplementary Tables

**Supplementary Table 1. MRI acquisition parameters.**

| **Parameter** | **3D T1-weighted MPRAGE** | **T2WI** | **3D T2 FLAIR** | **SWI** | **DTI** |
| --- | --- | --- | --- | --- | --- |
| Repetition time (msec) | 1500 | 3420 | 5000 | 28 | 6700 |
| Echo time (msec) | 1.9 | 105 | 201 | 20 | 72 |
| Flip angle (degree) | 9 | 150 | 120 | 15 | 90 |
| No. of signals acquired | 1 | 2 | 1 | 1 | 1 |
| Matrix | 256 ´ 256 | 448 ´ 406 | 256 ´ 244 | 448 ´ 225 | 128 ´ 128 |
| Section thickness (mm) | 1 | 4 | 1 | 2.5 | 2.5 |
| Intersection gap (mm) | 0 | 0.8 | 0 | 0 | 0 |
| Field of view (mm^2^) | 250 ´ 250 | 199 ´ 220 | 238 ´ 250 | 172 ´ 240 | 240 ´ 240 |
| Diffusion direction | NA | NA | NA | NA | 30 |
| b-value | NA | NA | NA | NA | 1000 s/mm^2^ |

Abbreviations: MPRAGE = Magnetization-Prepared Rapid Acquisition Gradient Echo, NA = Not Available.

**Supplementary Table 2. Groupwise comparison of regional log-transformed PVS across all pathological subgroups.**

|  | **BG PVS** | **Frontal PVS** | **Parietal PVS** | **Temporal PVS** | **Occipital PVS** |
| --- | --- | --- | --- | --- | --- |
| F (3,301) | 13.28 | 1.39 | 3.66 | 6.52 | 5.88 |
| p-value | **<0.0001****** | 0.247 | **0.013*** | **<0.001***** | **<0.001***** |
| Partial η² (90% CI) | 0.117 (0.060, 0.169) | 0.014 (0.000, 0.034) | 0.035 (0.004, 0.068) | 0.061 (0.019, 0.102) | 0.055 (0.016, 0.095) |

All PVS volumes are log-transformed.

All reported p-values are corrected using the Benjamini-Hochberg FDR procedure.

FDR-corrected significance: *p<0.05, **p<0.01, ***p<0.001, ****p<0.0001.

Abbreviations: BG = Basal Ganglia, PVS = Perivascular Spaces, FDR = False Discovery Rate, CI = Confidence Interval.

**Supplementary Table 3. Estimated marginal means of regional log-transformed PVS by AB×VB group.**

|  | **BG PVS** | **Frontal PVS** | **Parietal PVS** | **Temporal PVS** | **Occipital PVS** |
| --- | --- | --- | --- | --- | --- |
| AB− VB−  (95% CI) | -8.66  (-8.86, -8.45) | -8.54  (-8.81, -8.27) | -8.79  (-9.08, -8.51) | -9.67  (-9.94, -9.41) | -9.02  (-9.21, -8.83) |
| AB− VB+  (95% CI) | -8.01  (-8.13, -7.89) | -8.22  (-8.37, -8.06) | -8.30  (-8.46, -8.13) | -9.07  (-9.23, -8.92) | -8.58  (-8.68, -8.47) |
| AB+ VB−  (95% CI) | -8.58  (-8.76, -8.40) | -8.25  (-8.49, -8.02) | -8.24  (-8.49, -7.99) | -8.99  (-9.22, -8.76) | -8.62  (-8.79, -8.46) |
| AB+ VB+  (95% CI) | -8.15  (-8.27, -8.04) | -8.29  (-8.44, -8.14) | -8.39  (-8.55, -8.24) | -9.12  (-9.27, -8.98) | -8.59  (-8.70, -8.49) |

All PVS volumes are log-transformed.

Abbreviations: BG = Basal Ganglia, PVS = Perivascular Spaces, AB = Amyloid-β Burden, VB = Vascular Burden, CI = Confidence Interval.

**Supplementary Table 4. Conditional effects of AB and VB status from AB×VB interaction models predicting regional log-transformed PVS.**

|  | **BG PVS** | **Frontal PVS** | **Parietal PVS** | **Temporal PVS** | **Occipital PVS** |
| --- | --- | --- | --- | --- | --- |
| **AB effect among VB− participants** | | | | | |
| Estimated contrast  (95% CI) | 0.08  (-0.19, 0.34) | 0.28  (-0.06, 0.63) | 0.55  (0.19, 0.92) | 0.69  (0.35, 1.02) | 0.40  (0.16, 0.64) |
| p-value | 0.58 | 0.14 | **0.005**** | **<0.001***** | **0.003**** |
| **AB effect among VB+ participants** | | | | | |
| Estimated contrast  (95% CI) | -0.15  (-0.31, 0.02) | -0.08  (-0.29, 0.14) | -0.10  (-0.32, 0.12) | -0.05  (-0.28, 0.15) | -0.02  (-0.16, 0.13) |
| p-value | 0.38 | 0.75 | 0.75 | 0.75 | 0.83 |
| **VB effect among AB− participants** | | | | | |
| Estimated contrast  (95% CI) | 0.65  (0.40, 0.89) | 0.32  (0.001, 0.64) | 0.50  (0.16, 0.83) | 0.61  (0.30, 0.91) | 0.44  (0.22, 0.66) |
| p-value | **<0.0001****** | 0.049* | **0.004**** | **<0.001***** | **<0.001***** |
| **VB effect among AB+ participants** | | | | | |
| Estimated contrast  (95% CI) | 0.43  (0.20, 0.65) | -0.04  (-0.33, 0.25) | -0.15  (-0.46, 0.15) | -0.13  (-0.41, 0.14) | 0.03  (-0.17, 0.23) |
| p-value | **<0.001***** | 0.80 | 0.57 | 0.57 | 0.80 |

All PVS volumes are log-transformed.

All reported p-values are corrected using the Benjamini-Hochberg FDR procedure.

FDR-corrected significance: *p<0.05, **p<0.01, ***p<0.001, ****p<0.0001.

Abbreviations: BG = Basal Ganglia, PVS = Perivascular Spaces, AB = Amyloid-β Burden, VB = Vascular Burden, CI = Confidence Interval, FDR = False Discovery Rate.

**Supplementary Table 5. Multivariable linear regression results for regional log-transformed PVS (Same models as Supplementary Figure 2).**

| **Parameter** | **BG PVS** | **Frontal PVS** | **Parietal PVS** | **Temporal PVS** | **Occipital PVS** |
| --- | --- | --- | --- | --- | --- |
| **AB** | | | | | |
| β (95% CI) | 0.09 (−0.17, 0.35) | 0.23 (−0.12, 0.57) | 0.44 (0.07, 0.80) | 0.58 (0.25, 0.92) | 0.34 (0.10, 0.58) |
| p-value | 0.51 | 0.25 | **0.03*** | **0.004**** | **0.01*** |
| **CMB** | | | | | |
| β (95% CI) | 0.02 (−0.19, 0.23) | 0.16 (−0.11, 0.44) | 0.13 (−0.16, 0.42) | 0.08 (−0.19, 0.35) | 0.09 (−0.10, 0.28) |
| p-value | 0.83 | 0.63 | 0.63 | 0.70 | 0.63 |
| **Infarcts** | | | | | |
| β (95% CI) | 0.42 (0.16, 0.68) | 0.24 (−0.10, 0.58) | 0.26 (−0.10, 0.61) | 0.13 (−0.20, 0.46) | 0.07 (−0.16, 0.30) |
| p-value | **0.008**** | 0.28 | 0.28 | 0.55 | 0.56 |
| **WMH** | | | | | |
| β (95% CI) | 0.50 (0.27, 0.73) | 0.13 (−0.17, 0.44) | 0.26 (−0.06, 0.58) | 0.40 (0.10, 0.69) | 0.40 (0.19, 0.60) |
| p-value | **<0.001***** | 0.39 | 0.14 | **0.01*** | **<0.001***** |
| **CMB × AB** | | | | | |
| β (95% CI) | 0.01 (−0.27, 0.29) | −0.03 (−0.40, 0.35) | 0.002 (−0.38, 0.39) | 0.0001 (−0.36, 0.36) | 0.09 (−0.17, 0.34) |
| p-value | 1.00 | 1.00 | 1.00 | 1.00 | 1.00 |
| **Infarcts × AB** | | | | | |
| β (95% CI) | −0.03 (−0.38, 0.32) | −0.44 (−0.91, 0.02) | −0.58 (−1.06, −0.09) | −0.26 (−0.71, 0.19) | −0.01 (−0.32, 0.31) |
| p-value | 0.96 | 0.15 | 0.10 | 0.42 | 0.96 |
| **WMH × AB** | | | | | |
| β (95% CI) | −0.28 (−0.58, 0.02) | −0.16 (−0.57, 0.24) | −0.36 (−0.78, 0.06) | −0.58 (−0.97, −0.19) | −0.43 (−0.71, −0.15) |
| p-value | 0.11 | 0.42 | 0.11 | **0.009**** | **0.009**** |

All PVS volumes are log-transformed.

All reported p-values are corrected using the Benjamini-Hochberg FDR procedure.

FDR-corrected significance: *p<0.05, **p<0.01, ***p<0.001, ****p<0.0001.

Abbreviations: BG = Basal Ganglia, PVS = Perivascular Spaces, AB = Amyloid-β Burden, CMB = Cerebral Microbleed, WMH = White Matter Hyperintensity, CI = Confidence Interval, FDR = False Discovery Rate.

**Supplementary Table 6. Continuous SUVR multivariable linear regression of amyloid-vascular effects on regional PVS.**

| **Parameter** | **BG PVS** | **Frontal PVS** | **Parietal PVS** | **Temporal PVS** | **Occipital PVS** |
| --- | --- | --- | --- | --- | --- |
| **VB** | | | | | |
| β (95% CI) | 0.73 (0.29, 1.17) | 0.45 (−0.13, 1.02) | 0.82 (0.22, 1.42) | 1.01 (0.46, 1.55) | 0.79 (0.40, 1.18) |
| p-value | **0.002**** | 0.127 | **0.009**** | **<0.001***** | **<0.001***** |
| **SUVR** | | | | | |
| β (95% CI) | 0.01 (−0.20, 0.21) | 0.17 (−0.10, 0.44) | 0.39 (0.11, 0.67) | 0.49 (0.24, 0.75) | 0.32 (0.14, 0.51) |
| p-value | 0.941 | 0.270 | **0.011*** | **<0.001***** | **0.001**** |
| **SUVR × VB** | | | | | |
| β (95% CI) | −0.12 (−0.37, 0.12) | −0.20 (−0.52, 0.12) | −0.41 (−0.75, −0.08) | −0.49 (−0.80, −0.19) | −0.35 (−0.57, −0.13) |
| p-value | 0.316 | 0.286 | **0.026*** | **0.004**** | **0.004**** |

All PVS volumes are log-transformed.

All reported p-values are corrected using the Benjamini-Hochberg FDR procedure.

FDR-corrected significance: *p<0.05, **p<0.01, ***p<0.001, ****p<0.0001.

Abbreviations: BG = Basal Ganglia, PVS = Perivascular Spaces, AB = Amyloid-β Burden, VB = Vascular Burden, CI = Confidence Interval, FDR = False Discovery Rate.

# Supplementary Figures


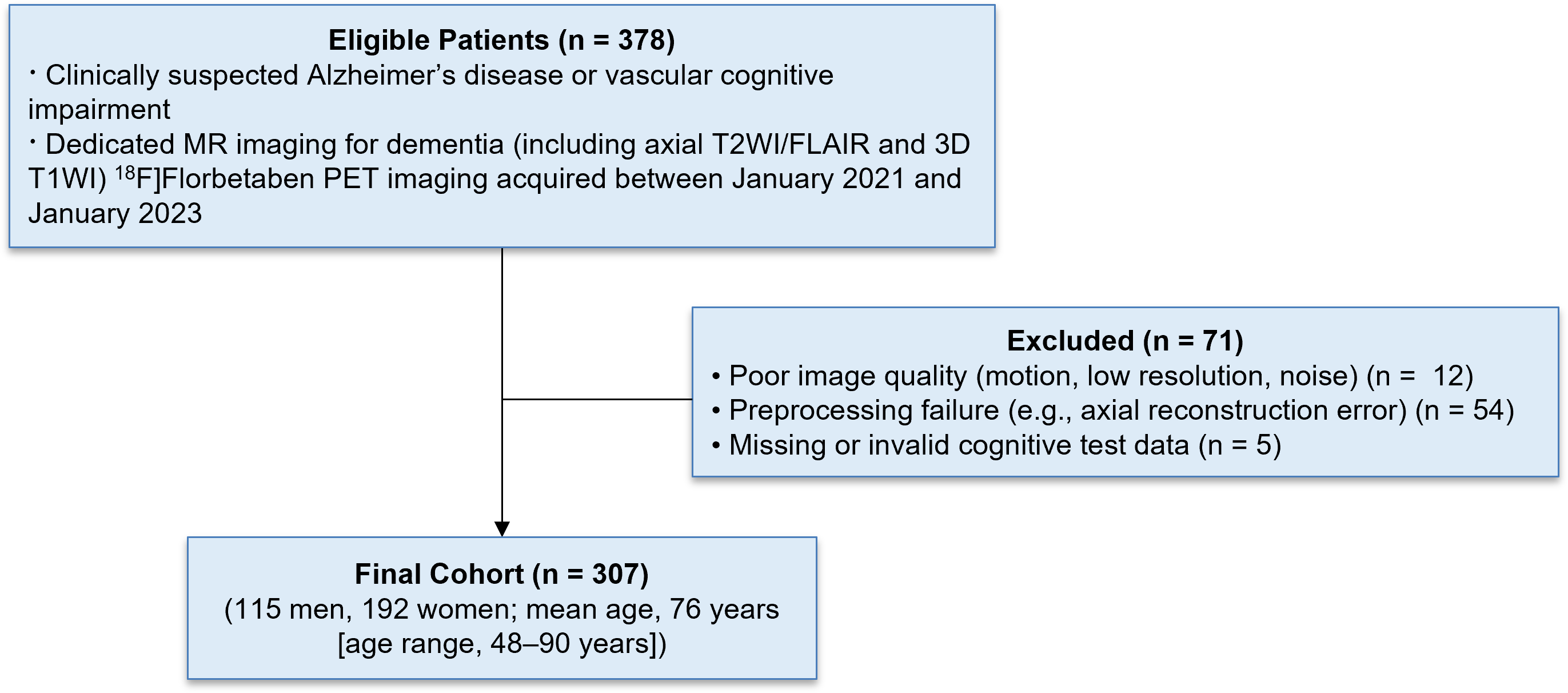


**Supplementary Figure 1. Study Participants’ Enlistment and Exclusion Criteria.**

Out of a total of 378 participants, subjects used in the final study were stratified based on visual quality of MRI scans, failure in axial-plane image reconstruction during MRI acquisition and preprocessing steps, and absence of standardized cognitive function test results.


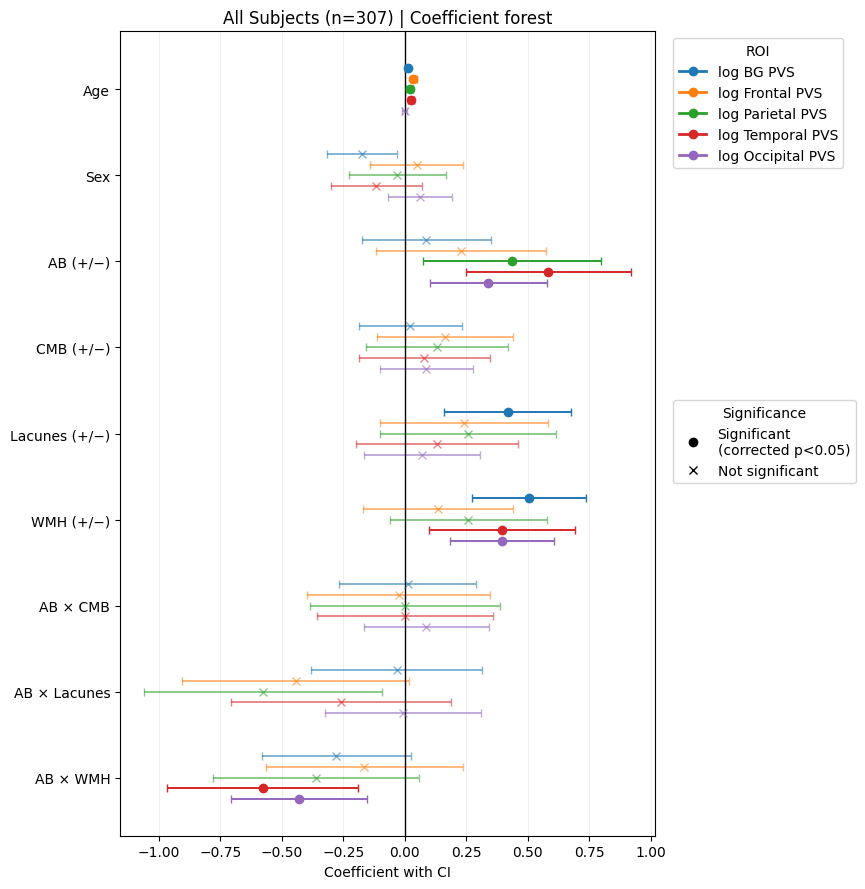


**Supplementary Figure 2. Regional Marker-Specific Amyloid-Vascular Interaction Effects on PVS.**

Forest plot of multivariable linear regression coefficients and 95% confidence intervals (CI) for models predicting regional log-transformed PVS‑VF from amyloid burden (AB) status, binary vascular marker status (CMB, lacunar infarcts, and WMH), and their interaction terms (AB×CMB, AB×Lacunes, and AB×WMH), adjusted for age and sex. CMB status was defined as positive if any CMB was present; infarct status as present versus absent; and WMH status as positive if PVWMH Fazekas ≥2 or DWMH Fazekas ≥2. Coefficients and CIs are color-coded by region. For each predictor, p-values were corrected across regions using BH-FDR procedure. Circles with thick CI bars indicate FDR-significant effects (corrected p<0.05); crosses with thin CI bars indicate non-significant effects.
